# Zn Supplementation Mitigates Drought Effects on Cotton by Improving Photosynthetic Performance and Antioxidant Defense Mechanisms

**DOI:** 10.3390/antiox12040854

**Published:** 2023-04-01

**Authors:** Touhidur Rahman Anik, Mohammad Golam Mostofa, Md. Mezanur Rahman, Md. Arifur Rahman Khan, Protik Kumar Ghosh, Sharmin Sultana, Ashim Kumar Das, Md. Saddam Hossain, Sanjida Sultana Keya, Md. Abiar Rahman, Nusrat Jahan, Aarti Gupta, Lam-Son Phan Tran

**Affiliations:** 1 Department of Plant and Soil Science, Institute of Genomics for Crop Abiotic Stress Tolerance, Texas Tech University, Lubbock, TX 79409, USA; 2 Department of Energy Plant Research Laboratory, Michigan State University, East Lansing, MI 48824, USA; 3 Department of Biochemistry and Molecular Biology, Michigan State University, East Lansing, MI 48824, USA; 4 Department of Agronomy, Bangabandhu Sheikh Mujibur Rahman Agricultural University, Gazipur 1706, Bangladesh; 5 Institute of Biotechnology and Genetic Engineering, Bangabandhu Sheikh Mujibur Rahman Agricultural University, Gazipur 1706, Bangladesh; 6 Department of Agroforestry and Environment, Bangabandhu Sheikh Mujibur Rahman Agricultural University, Gazipur 1706, Bangladesh; 7 Cotton Research Training and Seed Multiplication Farm, Cotton Development Board, Gazipur 1740, Bangladesh

**Keywords:** antioxidant enzymes, cotton, drought, photosynthesis, reactive oxygen species, zinc

## Abstract

Drought is recognized as a paramount threat to sustainable agricultural productivity. This threat has grown more severe in the age of global climate change. As a result, finding a long-term solution to increase plants’ tolerance to drought stress has been a key research focus. Applications of chemicals such as zinc (Zn) may provide a simpler, less time-consuming, and effective technique for boosting the plant’s resilience to drought. The present study gathers persuasive evidence on the potential roles of zinc sulphate (ZnSO_4_·7H_2_O; 1.0 g Kg^−1^ soil) and zinc oxide (ZnO; 1.0 g Kg^−1^ soil) in promoting tolerance of cotton plants exposed to drought at the first square stage, by exploring various physiological, morphological, and biochemical features. Soil supplementation of ZnSO_4_ or ZnO to cotton plants improved their shoot biomass, root dry weight, leaf area, photosynthetic performance, and water-use efficiency under drought stress. Zn application further reduced the drought-induced accumulations of H_2_O_2_ and malondialdehyde, and electrolyte leakage in stressed plants. Antioxidant assays revealed that Zn supplements, particularly ZnSO_4_, reduced reactive oxygen species (ROS) accumulation by increasing the activities of a range of ROS quenchers, such as catalase, ascorbate peroxidase, glutathione *S*-transferase, and guaiacol peroxidase, to protect the plants against ROS-induced oxidative damage during drought stress. Increased leaf relative water contents along with increased water-soluble protein contents may indicate the role of Zn in improving the plant’s water status under water-deficient conditions. The results of the current study also suggested that, in general, ZnSO_4_ supplementation more effectively increased cotton drought tolerance than ZnO supplementation, thereby suggesting ZnSO_4_ as a potential chemical to curtail drought-induced detrimental effects in water-limited soil conditions.

## 1. Introduction

Droughts are one of the most significant challenges in world agriculture, diminishing crop productivity and sustainability [1]. The situation has worsened in recent decades due to rapid and unprecedented variations in global climatic conditions [2]. A drought can disrupt plant life cycle by affecting their physiology, morphology, and overall metabolism at any stages of the plant’s growth and development. A drought can impede many physiological and biochemical processes, including photosynthesis, water and nutrient acquisition, osmotic balance, and reactive oxygen species (ROS) balance [3,4,5]. To fight against the negative consequences of droughts, plants adapt various strategies, such as: (i) the development of extensive root systems to forage water and nutrients, (ii) the shrinkage of the leaf area to reduce transpirational water loss, (iii) the accumulation of compatible solutes such as amino acids, soluble sugars and proteins, and polyamines to protect and stabilize cellular structures from osmotic effects, and (iv) the up-regulation of antioxidant defense mechanisms to alleviate oxidative stress [6,7,8,9].

Cotton (*Gossypium* spp.) is one of the most lucrative economic fiber crops, which is cultivated across the world, mainly in China, India, the United States, and Pakistan [6]. Despite it having a higher tolerance to abiotic stresses than other crops do, cotton’s productivity and fiber quality have been severely impacted by extreme environmental factors such as droughts [10,11]. It is estimated that the United States, the world’s third-largest cotton producer, has seen its huge cotton yield reduction by about 70% over the past four decades [12], whereas global cotton yield penalties may vary from 50 to 73% owing to the degree of drought stress [13]. Thus, the improvement of cotton growth and productivity in the face of current global drought severity is timely and crucial to maintaining a steady supply of raw materials for garment and textile industries.

To overcome drought-induced adverse effects on cotton, various biotechnological, along with traditional breeding approaches, are currently in practice [14,15,16]. However, the excessive cost, protracted experimentation time, and occasionally ethical barriers have rendered these technologies inaccessible to farmers in numerous countries. Due to these circumstances, the application of exogenous chemicals such as zinc (Zn) may serve as an alternative, simple, less time-consuming, and immediate technique for enhancing the plant’s resilience to the negative effects of droughts. Zn acts as a crucial factor for activating many biological processes, which ultimately boosts the plant’s growth potential and survival, particularly under stressed environments [17]. Its roles include its function as a cofactor of more than 300 enzymes, the regulation of gene expression and protein synthesis, phosphate and glucose metabolism, and the maintenance of structural and functional integrity of ribosomes [18,19]. Under drought stress, Zn application enhances seed germination, cell membrane integrity, the water status, osmolyte buildup, stomatal regulation, water-use efficiency, photosynthesis, and antioxidant defense mechanisms in plants [17]. Considering its significant benefits to the functions of plants, there has been a lot of interest in exploring different sources of Zn for improving abiotic stress tolerance of various agricultural crops. For instance, zinc sulfate (ZnSO_4_) application increased the tolerances of cotton (*G. hirsutum*) to heat [20], sunflower (*Helianthus annuus*) to drought [21], and of maize (*Zea mays*) [22], eggplant (*Solanum melongena*) [23], rice (*Oryza sativa*) [24], and tomato (*Solanum lycopersicum*) to salinity [25]. On the other hand, zinc oxide application in the form of bulk (ZnO) or nano particles (ZnO-NPs) increased the tolerances of wheat (*Triticum aestivum*) to heat [26], sunflower [27], rice [28], and wheat to droughts [29], and of tomato [30] and lupine (*Lupinus termis*) to salinity [31]. These reports highlight the versatile use of Zn in plant abiotic stress management. However, further investigations at the morphological and biochemical levels would be helpful to elucidate the underlying mechanisms behind Zn-mediated drought stress acclimatization in crop plants, especially in a comparative manner.

In the current study, we explored the comparative effects of two readily available Zn sources, namely ZnSO_4_ and ZnO, in mitigating drought effects on cotton plants, which has not been studied yet. As such, we investigated several physiological and biochemical mechanisms in the presence and absence of ZnSO_4_ or ZnO to drought-stressed cotton plants. Specifically, we evaluated the effectiveness of individually supplied ZnSO_4_ and ZnO in drought adaptations by recording: (1) growth and biomass production, (2) water statuses with respect to relative water contents (RWCs) and leaf succulence, (3) gas exchange parameters, (4) photosynthetic pigment levels, (5) ROS generation and membrane lipid peroxidation, (6) antioxidant enzyme activities, and (7) osmoprotectant accumulation in cotton plants under water-shortage conditions.

## 2. Materials and Methods

### 2.1. Plant Materials and Experimental Design

A high-yielding cotton (*G. hirsutum*) variety, cv. CB-12, was used to evaluate the effects of Zn supplementations on mitigating the damaging effects of drought stress. Cotton Research Training and Seed Multiplication Farm, Cotton Development Board, Gazipur, Bangladesh, provided the cotton seeds that were utilized in the study. During the experimental period, the temperatures varied from 10 °C to 35 °C, with humidity ranging from 51% to 98%. First, healthy seeds were treated for 20 min with a 5% (*v/v*) sodium hypochlorite solution prepared with 0.2% (*v/v*) Tween-20 for surface sterilization, and subsequently rinsed three times with distilled water (dH_2_O). The seeds were pre-soaked in dH_2_O for 8 h at room temperature under dark conditions to accelerate the germination process. Ten pre-soaked seeds were then sown in 2.5-L plastic pots holding 2.6 Kg of soil each. As a means of preparing the soil, the topsoil was combined with cow manure and sand in a weight-based ratio of 2:1:0.5. The pH of the soil was 6.7. The soil was treated with the well-known pesticide furadan (3.0 g Kg^−1^) to stop the spread of diseases that originate there. Prior to seed sowing, 1.0 g ZnSO_4_ (ZnSO_4_·7H_2_O, CAS Number: 7446-20-0, Merck, Germany) or ZnO (CAS Number: 1314-13-2, Merck, Germany) was mixed with per Kg of the potting soil. Appropriate doses of ZnSO_4_ and ZnO were selected based on a small-scale experiment (Supplementary Figure S1). Seven equally germinated seeds were kept in each pot. A second dose of Zn supplementation was directly applied to the potted soil after 30 days of seed sowing (five-leaf stage). Forty-day plants at the first-square stage were subjected to drought stress using the methodology of Rahman et al. [32]. Plants were subjected to drought stress by having the irrigation cut off for 6 days, whereas the control plants were provided with adequate water during the experiment. In brief, the whole experimental setup was consisted of six treatment groups: (i) well-watered control plants (WW), (ii) ZnSO_4_-supplemented well-watered control plants (ZnSO_4_), (iii) ZnO-supplemented well-watered control plants (ZnO), (iv) drought-stressed plants (DS), (v) ZnSO_4_-supplemented drought-stressed plants (ZnSO_4_ + DS), and (vi) ZnO-supplemented drought-stressed plants (ZnO + DS). All morphological, physiological, and biochemical analyses were executed on the 3rd leaves (from the bottom), which were freshly harvested from 46-day-old plants. The experiment was performed three times to confirm the precision of the findings.

### 2.2. Measurement of Growth-Related Attributes and Leaf Succulence

Three cotton plants were randomly chosen from each treatment to measure shoot length, shoot dry weight (SDW), and root dry weight (RDW). Shoot length (cm) was measured using a measuring scale from the soil contact point to the tip of the plant. To measure SDW and RDW (g), samples were placed into an envelope, followed by oven drying at 72 °C until the weight became constant before recording the weights using an electric balance. Leaf area (cm^2^) and leaf succulence were estimated using the 3rd leaf (counted from the bottom) following the formulae of Carleton and Foote [33] and Silveira et al. [34], respectively.

### 2.3. Estimation of Electrolyte Leakage and Leaf Relative Water Contents

Leaf electrolyte leakage and RWCs were measured following the methods described by Das et al. [35] and Rahman et al. [36], respectively.

### 2.4. Evaluation of the Gas Exchange Parameters

Under full sunlight, between 11:30 A.M. and 1:00 P.M., a transportable infrared gas analyzer system (LI-6400XT, LI-COR Inc., Lincoln, NE, USA) was employed to measure net photosynthetic rate (*P_n_*), leaf temperature (LT), stomatal conductance to H_2_O (*g_sw_*), and transpiration rate (*E*). Thereafter, intrinsic water-use efficiency (WUE_int_) was calculated by dividing *P_n_* by *g_sw_*, while instantaneous water-use efficiency (WUE_ins_) was calculated by dividing *P_n_* by *E*.

### 2.5. Determination of the Levels of Chlorophylls and Carotenoids

The contents of chlorophylls (Chl *a*, Chl *b*, and total Chls) and carotenoids (Cars) were determined in leaf extracts collected using 80% acetone, following the methods described by Lichtenthaler and Wellburn [37].

### 2.6. Measurement of Malondialdehyde and Hydrogen Peroxide Levels

Malondialdehyde (MDA) and hydrogen peroxide (H_2_O_2_) levels in cotton leaf tissues were measured using the methods outlined by Kim et al. [38] and Yu et al. [39], respectively.

### 2.7. Quantification of Antioxidant Enzyme Activities

Cotton fresh leaves were utilized in the preparation of enzyme extracts, and subsequently, the activities of antioxidant enzymes, including guaiacol peroxidase (GPOD; EC: 1.11.1.7), glutathione *S*-transferase (GST; EC: 2.5.1.18), catalase (CAT; EC 1.11.1.6), and ascorbate peroxidase (APX; EC: 1.11.1.11) were determined according to the procedures depicted by Rahman et al. [40].

### 2.8. Measurement of Proline and Water-Soluble Proteins Contents

The content of proline was measured using the acid-ninhydrin method as outlined by Bates et al. [41] with a slight modification. First, acid-ninhydrin was prepared by mixing 0.625 g of ninhydrin in 15 mL of glacial acetic acid and 10 mL of 6 M orthophosphoric acid. Around 0.1 g of fresh cotton leaves was homogenized in 2.5 mL of a 3% sulfosalicylic acid aqueous solution (extraction buffer), and then centrifuged at 12,000 rpm for 10 min. Afterward, 0.5 mL of the supernatant was mixed with 0.5 mL of acid-ninhydrin and 0.5 mL of glacial acetic in a glass tube and incubated in a water bath at 100 °C for 1 h, and subsequently kept in ice to terminate the reaction. The reactant solution was then mixed with 1.5 mL of toluene, vortexed for 10 s, and incubated at room temperature for 10 min. The absorbance of the upper light orange layer separated from the aqueous phase was measured at 520 nm. Toluene was used as a blank. The concentration of proline was estimated from a standard curve using the following formula provided by Bates et al. [41].

On the other hand, the Bradford protein assay was employed to quantify the water-soluble proteins (WSPs) content of the cotton leaf samples [42]. Total free amino acids content were determined following the protocol of Lee and Takahashi [43].

### 2.9. Statistical Analysis

Statistix version 10.0.0.9 software (Analytical Software, Tallahassee, FL, USA) was used to perform one-way analysis of variance (ANOVA) and a mean comparison test on the acquired data using the least significant difference (LSD) test at a significance level of *p* < 0.05. Significant differences among the treatments are represented by alphabetic letters. The experiment was performed three times, and each of them had three biological replicates per treatment, and similar results were obtained. Means and standard deviations (SDs) of three biological replicates for each treatment from one independent experiment are shown in the figures.

## 3. Results

### 3.1. Selection of the Best Dose of ZnSO_4_ and ZnO for Drought Alleviation

A noteworthy improvement in phenotypic appearance, such as less wilting, yellowing, and the drying of leaves, in ZnSO_4_ (1 and 1.5 g Kg^−1^ soil) and ZnO (1 and 1.5 g Kg^−1^ soil)-supplemented drought-stressed cotton plants was observed as compared with that of ‘DS’ plants (Supplementary Figure S1A,B). Drought stress significantly reduced the shoot length (by 7.8%) and SDW (by 35.4%) in ‘DS’ plants compared with that of ‘WW’ plants (Supplementary Figure S1C–F). On the other hand, ZnSO_4_-supplemented (0.25, 0.5, 1.0, 1.5, and 2.0 g Kg^−1^ soil) drought-stressed plants showed noteworthy improvements in shoot length (by 5.5, 6.1, 14.4, 11.4, and 10.9%) and SDW (by 9.0, 11.9, 28.9, 23.8, and 15.4%, respectively) as compared with those of ‘DS’ plants (Supplementary Figure S1C,D). Likewise, ZnSO_4_-supplemented (0.25, 0.5, 1.0, 1.5, and 2.0 g Kg^−1^ soil) well-watered plants also showed notable improvements in shoot height (by 3.4, 2.9, 11.5, 7.8, and 6.3%) and SDW (by 4.1, 10.3, 28.1, 17.7, and 16.2%, respectively) as compared with those of ‘WW’ plants (Supplementary Figure S1C,D). Interestingly, in case of ZnSO_4_, all shoot biomass-related parameters tended to gradually increase up to 1.0 g Kg^−1^ soil supplementation; thereafter, they showed a decreasing trend (Supplementary Figure S1C,D). On the other hand, 0.25, 0.5, 1.0, 1.5, and 2.0 g ZnO Kg^−1^ soil supplementation induced noteworthy improvements of shoot length (by 6.6, 5.2, 10.5, 10.0, and 9.6%) and SDW (by 2.9, 9.0, 15.4, 15.1, and 13.6%, respectively) as compared with those of ‘DS’ plants (Supplementary Figure S1E,F). However, only 1.0, 1.5, and 2.0 g Kg^−1^ soil ZnO-supplemented well-watered plants showed notable improvements in shoot height (by 6.7, 5.9, and 6.3%) and SDW (by 13.6, 12.4, and 14.7%, respectively) as compared with those of ‘WW’ plants (Supplementary Figure S1E,F). In case of ZnO, all shoot biomass-related parameters under drought stress conditions tended to gradually increase up to 1.0 g Kg^−1^ soil supplementation, and afterwards, they showed a decreasing trend (Supplementary Figure S1E,F). Considering the above findings, the 1.0 g Kg^−1^ soil dose for both chemicals was chosen to carry out further physiological and biochemical studies.

### 3.2. Both ZnSO_4_ and ZnO Improved the Phenotypes, Growth, and Biomass of Cotton Plants Subjected to Drought Stress

**Figure 1:**
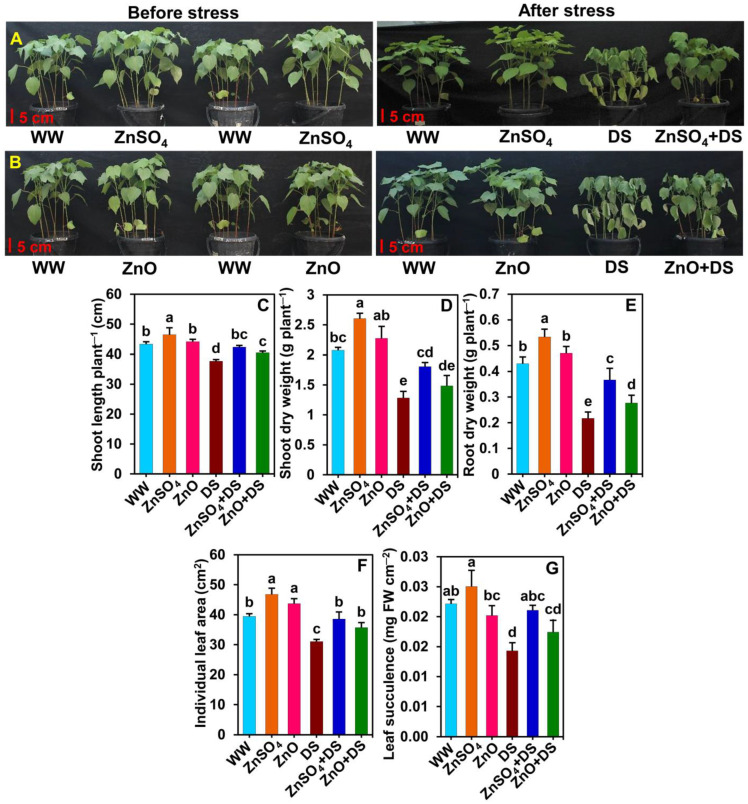
Effect of separately applied ZnSO_4_ (1.0 g Kg^−1^ soil) and ZnO (1.0 g Kg^−1^ soil) on cotton plants that were subjected to water deprivation-induced drought stress for 6 days. (**A**,**B**) Cotton plants were photographed before and after being subjected to drought stress. (**C**) Shoot length, (**D**) shoot dry weight, (**E**) root dry weight, (**F**) individual leaf area, and (**G**) leaf succulence of cotton plants under different treatments. The means and standard deviations (*n* = 3) are displayed as bars. Significant changes (*p* < 0.05) among the treatments are denoted by different letters (a–e) above the bars as calculated using the least significant difference test. Here, the treatments are well-watered plants (WW), ZnSO_4_-supplemented well-watered plants (ZnSO_4_), ZnO-supplemented well-watered plants (ZnO), drought-stressed plants (DS), ZnSO_4_-supplemented drought-stressed plants (ZnSO_4_ + DS), and ZnO-supplemented drought-stressed plants (ZnO + DS). FW, fresh weight.

Water deprivation for 6 days caused remarkable disturbances in the phenotypic appearance of ‘DS’ plants as compared with that of ‘WW’ plants (Figure 1A,B). Typical drought-stressed symptoms such as wilting, the yellowing of leaves, and the drying of the bottom leaves were evidently observed in ‘DS’ plants (Figure 1A,B). Contrariwise, a noteworthy improvement in phenotypes such as less wilting and the yellowing of leaves were observed in ‘ZnSO_4_ + DS’ and ‘ZnO + DS’ plants (Figure 1A,B). Significant reductions of shoot length (by 13.1%), SDW (by 38.2%), RDW (by 49.6%), leaf area (by 21.2%), and leaf succulence (by 35.4%) were demonstrated in ‘DS’ plants compared with the respective values of ‘WW’ plants (Figure 1C–G). On the other hand, ZnSO_4_ and ZnO supplementations resulted in significant enhancements of the shoot length (by 12.4 and 7.5%), SDW (by 40.5%, only in ‘ZnSO_4_ + DS’ plants), RDW (by 69.2 and 27.7%), leaf area (by 24.2 and 15.0%), and leaf succulence (by 47.2%, only in ‘ZnSO_4_ + DS’ plants) in ‘ZnSO_4_ + DS’ and ‘ZnO + DS’ plants, respectively, which contrast with those of ‘DS’ plants (Figure 1C–G). Likewise, soil supplementation with ZnSO_4_ significantly increased the shoot length (by 7.3%), SDW (by 25.4%), RDW (by 24.0%), and leaf area (by 18.8%) in ‘ZnSO_4_’ plants as compared with those of ‘WW’ plants (Figure 1C–F). However, in the case of ZnO supplementation, only leaf area was found to increase significantly by 10.8% in ‘ZnO’ plants, whereas shoot length, SDW, and RDW increased in a nonsignificant manner compared with those of ‘WW’ plants (Figure 1C–F). Furthermore, the leaf succulence values of ‘WW’, ‘ZnSO_4_’, and ‘ZnO’ plants were comparable (Figure 1G). In general, plants supplemented with ZnSO_4_ had a better phenotypic appearance, as well as better growth and biomass outputs, than those of ZnO-supplemented plants in both stressed and non-stressed circumstances.

### 3.3. Both ZnSO_4_ and ZnO Improved Gas Exchange Attributes of Cotton Plants Subjected to Drought Stress

**Figure 2:**
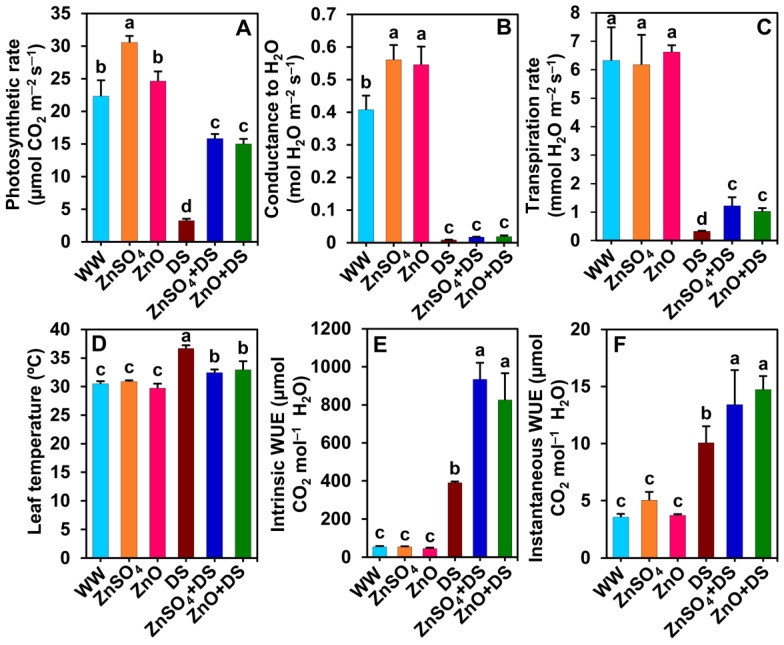
Effect of separately applied ZnSO_4_ (1.0 g Kg^−1^ soil) and ZnO (1.0 g Kg^−1^ soil) on (**A**) photosynthetic rate, (**B**) stomatal conductance to H_2_O, (**C**) transpiration rate, (**D**) leaf temperature, (**E**) intrinsic WUE, and (**F**) instantaneous WUE of cotton plants that were subjected to water deprivation-induced drought stress for 6 days. The means and standard deviations (*n* = 3) are displayed as bars. Significant changes (*p* < 0.05) among the treatments are denoted by different letters (a–d) above the bars as calculated using the least significant difference test. Here, the treatments are well-watered plants (WW), ZnSO_4_-supplemented well-watered plants (ZnSO_4_), ZnO-supplemented well-watered plants (ZnO), drought-stressed plants (DS), ZnSO_4_-supplemented drought-stressed plants (ZnSO_4_ + DS), and ZnO-supplemented drought-stressed plants (ZnO + DS). FW, fresh weight; WUE, water-use efficiency.

Significant reductions of *P_n_* (by 85.5%), *g_sw_* (by 98.0%), and *E* (by 95.0%) were observed in ‘DS’ plants, which contrast with the respective values of ‘WW’ plants (Figure 2A–C). On the other hand, notable increments of LT (by 20.4%), WUE_int_ (by 612.1%), and WUE_ins_ (by 183.0%) were observed in ‘DS’ plants as compared with the respective values of ‘WW’ plants (Figure 2D–F). Interestingly, significant increases in *P_n_* (by 389.3 and 364.2%), *E* (by 279.2 and 217.5%), WUE_int_ (by 139.2 and 111.6%), and WUE_ins_ (by 33.2 and 46.2%) and a reduction of LT (by 11.6 and 10.3%) were evident in ‘ZnSO_4_ + DS’ and ‘ZnO + DS’ plants, respectively, as contrasted with the respective values of ‘DS’ plants (Figure 2A,C–F). A non-significant improvement of *g_sw_* (by 105.3 and 124.2%) was recorded in ‘ZnSO_4_ + DS’ and ‘ZnO + DS’ plants, respectively, compared with that of ‘DS’ plants (Figure 2B). Nonetheless, significant increases in *P_n_* (by 36.8%, only in ‘ZnSO_4_’ plants) and *g_sw_* (by 37.5 and 33.8%) were recorded in ‘ZnSO_4_’ and ‘ZnO’ plants as compared with the respective values of ‘WW’ plants (Figure 2A,B). However, non-significant variations in *E*, LT, WUE_int_, and WUE_ins_ were observed among ‘WW’, ‘ZnSO_4_’, and ‘ZnO’ plants (Figure 2C–F).

### 3.4. Both ZnSO_4_ and ZnO Improved Photosynthetic Pigment Contents of Cotton Plants Subjected to Drought Stress

**Figure 3:**
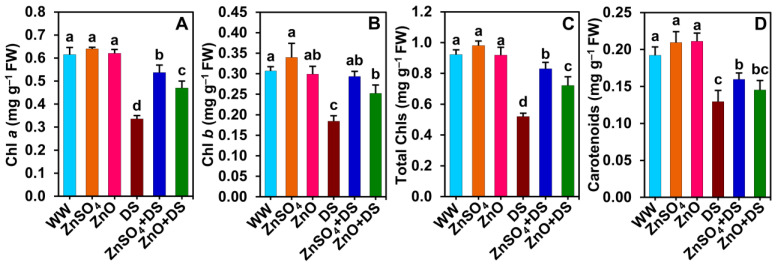
Effect of separately applied ZnSO_4_ (1.0 g Kg^−1^ soil) and ZnO (1.0 g Kg^−1^ soil) on the content of (**A**) Chl *a*, (**B**) Chl *b*, (**C**) total Chls, and (**D**) carotenoids in the leaves of cotton plants that were subjected to water deprivation-induced drought stress for 6 days. The means and standard deviations (*n* = 3) are displayed as bars. Significant changes (*p* < 0.05) among the treatments are denoted by different letters (a–d) above the bars as calculated using the least significant difference test. Here, the treatments are well-watered plants (WW), ZnSO_4_-supplemented well-watered plants (ZnSO_4_), ZnO-supplemented well-watered plants (ZnO), drought-stressed plants (DS), ZnSO_4_-supplemented drought-stressed plants (ZnSO_4_ + DS), and ZnO-supplemented drought-stressed plants (ZnO + DS). Chl, chlorophyll; FW, fresh weight.

Expectedly, significantly reduced levels of Chl *a*, Chl *b*, total Chls, and Cars were recorded in the leaves of ‘DS’ plants relative to those of ‘WW’ plants (Figure 3A–D). On the other hand, substantial increases in the contents of Chl *a* (by 60.0 and 40.0%), Chl *b* (by 59.2 and 37.0%), total Chls (by 59.7 and 38.9%, respectively), and Cars (by 23.3, only in ‘ZnSO_4_ + DS’ plants) were observed in ‘ZnSO_4_ + DS’ and ‘ZnO + DS’ plants compared with those values in ‘DS’ plants (Figure 3A–D). However, the levels of Chl *a*, Chl *b* (only in ‘ZnSO_4_’ plants), total Chls, and Cars showed nonsignificant improvements in ‘ZnSO_4_’ and ‘ZnO’ plants compared to the respective values in ‘WW’ plants (Figure 3A–D).

### 3.5. Both ZnSO_4_ and ZnO Protected Cotton Plants from Drought-Induced Oxidative Damage

**Figure 4:**
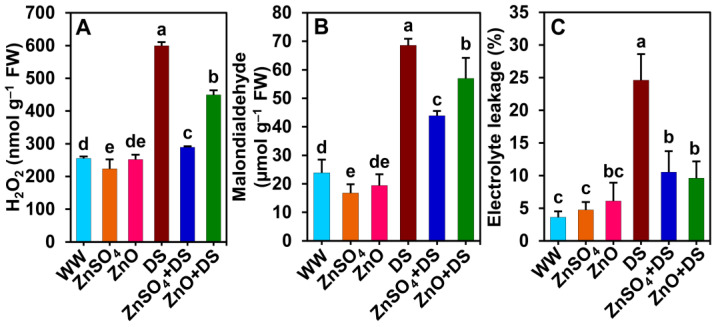
Effect of separately applied ZnSO_4_ (1.0 g Kg^−1^ soil) and ZnO (1.0 g Kg^−1^ soil) on (**A**) hydrogen peroxide (H_2_O_2_), (**B**) malondialdehyde, and (**C**) electrolyte leakage levels in the leaves of cotton plants that were subjected to water deprivation-induced drought stress for 6 days. The means and standard deviations (*n* = 3) are displayed as bars. Significant changes (*p* < 0.05) among the treatments are denoted by different letters (a–e) above the bars as calculated using the least significant difference test. Here, the treatments are well-watered plants (WW), ZnSO_4_-supplemented well-watered plants (ZnSO_4_), ZnO-supplemented well-watered plants (ZnO), drought-stressed plants (DS), ZnSO_4_-supplemented drought-stressed plants (ZnSO_4_ + DS), and ZnO-supplemented drought-stressed plants (ZnO + DS). FW, fresh weight.

In comparison with ‘WW’ plants, water withholding for 6 days caused significant increases in the levels of H_2_O_2_ (by 135.2%), MDA (187.3%), and EL (579.9%) in the leaves of ‘DS’ plants (Figure 4A–C). Conversely, ‘ZnSO_4_ + DS’ and ‘ZnO + DS’ plants displayed significantly lower levels of H_2_O_2_ (by 51.7 and 25.1%), MDA (by 36.0 and 17.0%), and EL (by 57.2 and 61.0%, respectively) compared with those of ‘DS’ plants (Figure 4A–C). Furthermore, compared with the ‘WW’ plants, significantly reduced levels of H_2_O_2_ (by 12.5%) and MDA (by 29.8%) were recorded in ‘ZnSO_4_’ plants, while the EL levels in ‘WW’ and ‘ZnSO_4_’ plants were comparable (Figure 4A–C). There was no statistically significant difference in the H_2_O_2_, MDA, or EL levels between the ‘ZnO’ and ‘WW’ plants (Figure 4A–C).

### 3.6. Both ZnSO_4_ and ZnO Boosted Antioxidant Enzyme Activities in Cotton Plants Subjected to Drought Stress

**Figure 5:**
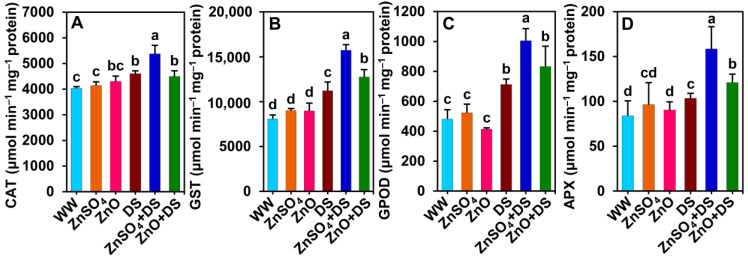
Effect of separately applied ZnSO_4_ (1.0 g Kg^−1^ soil) and ZnO (1.0 g Kg^−1^ soil) on the activities of (**A**) catalase (CAT), (**B**) glutathione *S*-transferase (GST), (**C**) guaiacol peroxidase (GPOD), and (**D**) ascorbate peroxidase (APX) in the leaves of cotton plants that were subjected to water deprivation-induced drought stress for 6 days. The means and standard deviations (*n* = 3) are displayed as bars. Significant changes (*p* < 0.05) among the treatments are denoted by different letters (a–d) above the bars as calculated using the least significant difference test. Here, the treatments are well-watered plants (WW), ZnSO_4_-supplemented well-watered plants (ZnSO_4_), ZnO-supplemented well-watered plants (ZnO), drought-stressed plants (DS), ZnSO_4_-supplemented drought-stressed plants (ZnSO_4_ + DS), and ZnO-supplemented drought-stressed plants (ZnO + DS).

Drought stress significantly increased the activity of antioxidant enzymes such as CAT (by 14.0%), GST (38.7%), GPOD (48.0%), and APX (23.2%) in ‘DS’ plants, which contrast with the respective values of ‘WW’ plants (Figure 5A–D). Individually supplied ZnSO_4_ and ZnO further significantly increased the activities of CAT (by 16.9%, only in ‘ZnSO_4_ + DS’ plants), GST (by 40.3 and 13.7%), GPOD (by 41.2%, only in ‘ZnSO_4_ + DS’ plants), and APX (by 53.6 and 16.9%) in ‘ZnSO_4_ + DS’ and ‘ZnO + DS’ plants, respectively, as compared with the respective values of ‘DS’ plants (Figure 5A–D). However, CAT, GST, GPOD, and APX activities remained comparable among the ‘WW’, ‘ZnSO_4_ + DS’, and ‘ZnO + DS’ plants (Figure 5A–D).

### 3.7. Both ZnSO_4_ and ZnO Improved Water Balance and Osmoprotectant Levels

**Figure 6:**
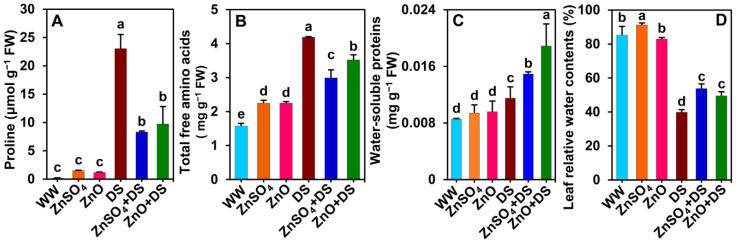
Effect of separately applied ZnSO_4_ (1.0 g Kg^−1^ soil) and ZnO (1.0 g Kg^−1^ soil) on the level of (**A**) proline, (**B**) total free amino acids, and (**C**) water-soluble proteins, as well as on the (**D**) relative water contents in the leaves of cotton plants that were subjected to water deprivation-induced drought stress for 6 days. The means and standard deviations (*n* = 3) are displayed as bars. Significant changes (*p* < 0.05) among the treatments are denoted by different letters (a–e) above the bars as calculated using the least significant difference test. Here, the treatments are well-watered plants (WW), ZnSO_4_-supplemented well-watered plants (ZnSO_4_), ZnO-supplemented well-watered plants (ZnO), drought-stressed plants (DS), ZnSO_4_-supplemented drought-stressed plants (ZnSO_4_ + DS), and ZnO-supplemented drought-stressed plants (ZnO + DS). FW, fresh weight.

Under drought stress, ‘DS’ plants showed remarkable increases in the content of proline (by 12,250.8%), total free amino acids (166.3%), and WSPs (34.6%) as compared with those of ‘WW’ plants (Figure 6A–C). Contrariwise, RWCs were found to be significantly reduced in ‘DS’ plants in comparison with those in ‘WW’ plants (Figure 6D). On the other hand, the Zn application significantly decreased the proline (by 64.0 and 57.7%) and total free amino acid (by 28.6 and 15.9%) levels, but increased the WSP (by 29.7 and 64.0%, respectively) contents in ‘ZnSO_4_ + DS’ and ‘ZnO + DS’ plants relative to those of ‘WW’ plants (Figure 6A–C). Interestingly, the RWCs level were found to be significantly increased in ‘ZnSO_4_ + DS’ and ‘ZnO + DS’ plants compared with the respective values in ‘DS’ plants (Figure 6D). Although the proline and WSP contents remained comparable among ‘WW’, ‘ZnSO_4_ + DS’, and ‘ZnO + DS’ plants, the total free amino acid contents (by 43.0 and 42.8%) and RWCs (by 7.1%) significantly increased in ‘ZnSO_4_’ and ‘ZnO’ plants, respectively, compared with the respective values in ‘WW’ plants (Figure 6A–D).

## 4. Discussion

The current study provides a picture of how Zn application, either in the form of ZnSO_4_ or ZnO, confronted the deleterious effects of drought in cotton plants applied at the first square stage. To understand Zn-mediated drought tolerance mechanisms, we explored various morphological and biochemical features that are crucial for cotton survival in water-limited circumstances.

Drought imposition induced severe damage to cotton plants, as seen by the withering, yellowing, and drying of leaves, as well as the considerable slowing of growth and biomass gain (Figure 1A–F). Zn supplementations, on the other hand, reduced leaf wilting and yellowing symptoms and increased growth and biomass production, thus helping cotton plants overcome the drought-induced negative effects (Figure 1A–F). Importantly, the increased root biomass in Zn-supplemented plants indicated that Zn enabled cotton plants to forage more water and nutrients, which ultimately resulted in enhanced photosynthetic activity and improved growth characteristics in drought-stricken environments (Figure 1E) [44,45]. The notable improvements in cotton growth and the shoot and root biomass of Zn-supplemented plants supported that exogenous Zn played a crucial role in enhancing cotton’s resilience to drought stress. Improvements of the plant’s growth and biomass under water-stress conditions upon the application of ZnO [27] and ZnSO_4_ [21] were also reported in *H. annuus*. Interestingly, the current study found that ZnSO_4_-supplemented plants thrived and generated more shoot and root biomass than ZnO-supplemented plants did in both stress and non-stress situations (Figure 1A–F). A widespread agreement exists that soil pH is a major determinant of Zn availability, especially when it is applied in a non-soluble form such as ZnO [46,47,48]. In general, soil acidity increases ZnO solubility and availability [48]. On the other hand, a previous study showed that when Zn was administered as ZnSO_4_ (the ionic form), it could be dissolved across a wide range of pH values (4.10–7.98) [49]. The soil used in the current study could be classified as neutral because the soil pH was 6.7. This could be a limiting factor that reduced Zn availability to the plants supplemented with ZnO. It could be likely that the higher solubility and better distribution nature of ZnSO_4_ compared to those of ZnO might ensure increased availability of Zn to soil; thus, ZnSO_4_ performed better in improving cotton’s growth performance and drought tolerance (Figure 1A–F).

Water stress undermines crop growth and productivity by impairing the leaf water status and gas exchange ability of plants [50]. Drought stress reduces the stomatal conductance and transpiration rates, resulting in higher leaf temperature and wilting [51], as also demonstrated in the current study (Figure 1A,B, and Figure 2D). It is well known that Zn improves stomatal conductance by maintaining K^+^ influx in guard cells, resulting in higher WUE in water-stressed conditions [33]. In line with this statement, noteworthy improvements in stomatal conductance, the transpiration rate, and WUE were observed in ‘ZnSO_4_ + DS’ and ‘ZnO + DS’ plants, indicating a better drought acclimation strategy in Zn-supplied cotton plants (Figure 2B,C,E,F).

Reduced growth and biomass production (Figure 1A–F) can be attributed partly to drought-induced impairment of the photosynthetic performance (Figure 2A), possibly through the degradation of the photosynthetic pigments (Figure 3A–D), as also observed in *Glycine max* and *Oudeneya africana* under water-shortage conditions [52,53]. Conversely, Zn-supplemented cotton plants retained higher levels of photosynthetic pigments (e.g., Chl *a*, Chl *b*, total Chls, and Cars), along with a heightened photosynthetic rate, upon their exposure to water-withholding-induced drought stress (Figure 2A and Figure 3A–D). These findings suggested that Zn might contribute to the synthesis and/or slowing of the destruction of photosynthetic pigments, thereby aiding in the maintenance of optimal photosynthetic performance under water-stressed conditions (Figure 2A and Figure 3A–D). Indeed, Zn acts as a co-factor for many proteins and enzymes involved in plant pigment biosynthesis, which is required to keep the chlorophyll biosynthesis pathway active, particularly in stressful environments [54]. The beneficial effects of Zn application in augmenting photosynthetic pigments and photosynthetic efficiency have also been well documented in a range of plant species, including *S. lycopersicum*, *T. aestivum*, and *Cicer arietinum* [55,56,57]. Previous reports suggest that there is a direct relationship between higher net photosynthesis and higher leaf area [33], which was also evidently observed in Zn-supplemented cotton plants (Figure 1F). Leaf area expansion directly regulates the light acquisition rate of plants, and thus, the overall photosynthetic performance and carbon assimilation and distribution in different plant parts [58,59,60]. Together, Zn supplementation improved the gas exchange characteristics and safeguarded photosynthetic pigments, which enhanced WUE and photosynthetic efficiency to support better growth and survival in a water-scarce environment.

Drought-induced ROS accumulation is thought to have a correlation with photo-oxidative damage and impairment of the plant’s growth [61,62], as was also clearly found in drought-stressed cotton plants (Figure 1A,B and Figure 4A). Our analyses showed that both ‘ZnSO_4_ + DS’ and ‘ZnO + DS’ plants generated less H_2_O_2_, and consequently, reduced levels of MDA and EL under water-deficit conditions (Figure 4A–C). Plants experiencing drought are more prone to lipid peroxidation, which results in the membrane damage responsible for a number metabolic disturbance at the cellular level [63]. Zn is special because it does not go through redox cycling while it is in the divalent state, which allows it to survive and be stable in biological media [64]. These characteristics allow Zn to shield membrane lipids from ROS toxicity, which in turn prevents ion leakage through the membranes [17]. Moreover, Zn addition remarkably enhanced the activities of several key antioxidant enzymes, namely CAT, GST, GPOD, and APX, in drought-stressed cotton plants (Figure 5A–D), suggesting a crucial role of Zn in protecting cells from oxidative damage by eliminating excessive H_2_O_2_ (Figure 4A and Figure 5A,C,D). The enhanced activity of GST in Zn-supplemented drought-stressed cotton plants (Figure 5B) might activate the GSH-dependent peroxide quenching mechanism to provide better protection against the drought-induced organic peroxides, reactive aldehydes, and lipid hydroperoxides [65]. In general, plants treated with ZnSO_4_ have higher levels of antioxidant activity than plants supplied with ZnO do, which is correlated with substantially lower buildup of H_2_O_2_ and MDA in ‘ZnSO_4_ + DS’ plants compared to that of ‘ZnO + DS’ plants (Figure 4A and Figure 5A–D). This might be another reason for ZnSO_4_-treated plants’ improved drought acclimatization responses. In support of the current study, higher levels of antioxidant activities upon ZnSO_4_ application were also reported in *G. hirsutum* under heat stress [20], in *H. annuus* under drought stress [21], and in *S. melongena* under salinity stress [23].

**Figure 7:**
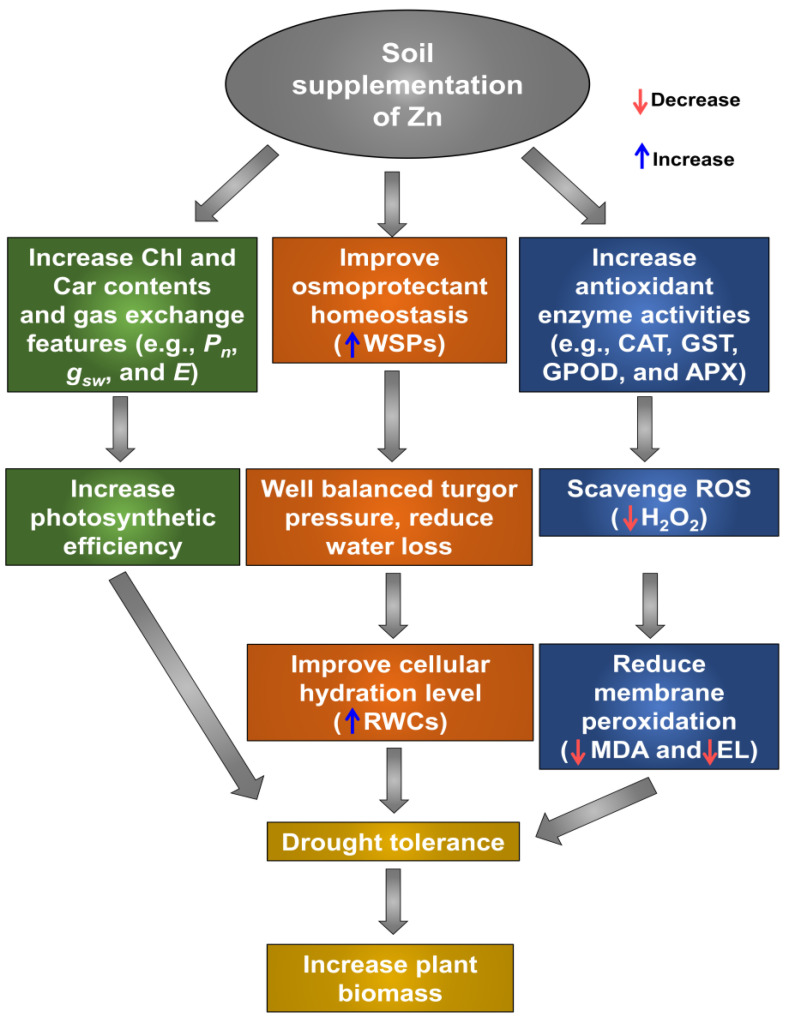
Function of zinc (Zn) in regulating cotton physiological and biochemical mechanisms to overcome drought-caused negative effects. Zn supplementation (either in the form of ZnSO_4_ or ZnO) to drought-stressed cotton plants significantly decreased their growth penalty, partly by protecting photosynthetic pigments and improving gas exchange features, which finally enhanced the overall photosynthetic efficiency and growth performance. Zn addition also activated the antioxidant defense mechanism by increasing the activities of enzymatic antioxidants (e.g., CAT, GST, GPOD, and APX), which helped protect Zn-supplemented cotton plants against reactive oxygen species (ROS)-induced oxidative stress and membrane damage. Furthermore, through boosting water-soluble proteins (WSPs) content, Zn aided osmoprotection, which eventually helped preserve leaf water status for osmotic adjustment under water-scarce conditions. Blue arrow represents an upward trend, while red arrow denotes a declining trend. APX, ascorbate peroxidase; Cars, carotenoids; CAT, catalase; Chls, chlorophylls; *E*, transpiration rate; EL, electrolyte leakage; GST, glutathione *S*-transferase; GPOD, guaiacol peroxidase; *g_sw_*, stomatal conductance to H_2_O; H_2_O_2_, hydrogen peroxide; MDA, malondialdehyde; *P_n_*, net photosynthetic rate; RWCs, relative water contents; Zn, zinc.

Under drought stress, plants accumulate a range of osmoprotectants, predominantly proline, to carry out osmotic adjustment [66]. Our observation indicated that despite the high accumulation of proline, proper RWCs were not maintained in ‘DS’ plants (Figure 6A,D). Likewise, when we were directly comparing them with ‘DS’ plants, the proline content was negatively correlated with the RWCs in ‘ZnSO_4_ + DS’ and ‘ZnO + DS’ plants, suggesting that Zn might contribute to replenishing water loss without significantly accumulating proline (Figure 6A). Thus, our study supported that the level of proline accumulation was correlated with the severity of cellular dehydration in cotton [67,68]. The extensive accumulation of free amino acids has usually been observed in response to different abiotic stresses, including drought (Figure 6B) [69]. An inadequate carbon supply due to reduced photosynthesis under drought stress provokes the accumulation of free amino acids (Figure 2A and Figure 6B), which can be used as alternative substrates for mitochondrial respiration [70]. However, increased photosynthetic efficiency may revert the cellular metabolism to more favorable growth conditions and reduce the accumulation of free amino acids [71], as was evident in ‘ZnSO_4_ + DS’ and ‘ZnO + DS’ plants in the current study (Figure 2A and Figure 6B). Alternately, Zn-supplemented plants might have used more free amino acids to increase their biomass even under drought conditions (Figure 1A–F), and as a result, there was lower accumulation of free amino acids in the plants (Figure 6B). Under water-deficit conditions, plants accumulate different low-molecular-weight substances, including WSPs, to maintain the osmotic balance [72]. Moreover, the role of Zn in improving protein synthesis is well recognized [17], as we expectedly recorded in ‘ZnSO_4_ + DS’ and ‘ZnO + DS’ plants (Figure 6C). The increased accumulation of WSPs may aid plants in keeping the leaf turgor pressure and stomatal conductance favorable for efficient CO_2_ absorption and boosting the capacity of the plant’s roots to effectively harness water [73]. A generalized mechanism of Zn-induced drought tolerance in cotton is presented in Figure 7.

## 5. Conclusions

The current study revealed the important roles of Zn in the modulation of physiological and biochemical defense mechanisms that assist cotton plants to overcome water-shortage-induced negative consequences (Figure 7). When they are contrasted with Zn-non-supplemented plants alone, Zn-supplemented cotton plants exhibited a greater root biomass, which might contribute to the acquisition of more water and nutrients for maintaining a better water status and photosynthetic performance under drought conditions (Figure 1E and Figure 2A). Zn application also radically reduced ROS accumulation, along with curtailing MDA and EL levels (Figure 4A–C), suggesting that Zn might play a key role in reversing ROS-induced oxidative damage to support cotton plants’ survival through drought stress. These results were further supported by augmented activities of enzymatic antioxidants, including CAT, GST, GPOD, and APX, which have important implications in ROS elimination (Figure 5A–D). Increased levels of WSPs upon Zn supplementation also assisted the water-stressed cotton plants to improve the water status and maintain the optimum osmotic balance (Figure 6C,D). This study also pointed out that ZnSO_4_ supplementation improved cotton’s drought tolerance compared with that caused by ZnO supplementation. One important reason for this observation may be the neutral pH of the soil used in this study, which might prevent ZnO from dissolving entirely, making it inaccessible to plants. It is also plausible that the dissociation of ZnSO_4_ provided sulfur, which could potentiate the formation of small defense molecules such as glutathione for conferring an extra protection layer against drought stress. Altogether, our results suggest that Zn acted as an imperative regulator of several morphological and biochemical processes, which increased the resilience of cotton plants toward drought stress. Moreover, our observations provide important clues at the physiological and biochemical levels that could be taken into account for further evaluation of Zn-regulated drought tolerance networks at the molecular level in cotton plants. Finally, additional research, including field tests and cost–benefit analyses, should be conducted to confirm that Zn application is a successful strategy for minimizing the negative effects of drought and lowering the cotton yield losses in soils with a limited water supply.

## Data Availability

Data are contained within the article and Supplementary Materials.

## Supplementary Materials

The following supporting information can be downloaded at: https://www.mdpi.com/article/10.3390/antiox12040854/s1, Figure S1. Effect of separately applied different doses of ZnSO_4_ (0.25, 0.5, 1.0, 1.5, and 2.0 g Kg^−1^ soil) and ZnO (0.25, 0.5, 1.0, 1.5, and 2.0 g Kg^−1^ soil) on cotton plants that were subjected to water deprivation-induced drought stress for 6 days.
