# Supplementary material for: Zn Supplementation Mitigates Drought Effects on Cotton by Improving Photosynthetic Performance and Antioxidant Defense Mechanisms
Source: Antioxidants (Basel). 2023 Apr 1;12(4):854. doi: 10.3390/antiox12040854 (PMC10135281; doi:10.3390/antiox12040854)
Supplement: Supplementary file 1 [file antioxidants-12-00854-s001.zip › antioxidants-2244933-supplementary.pdf]

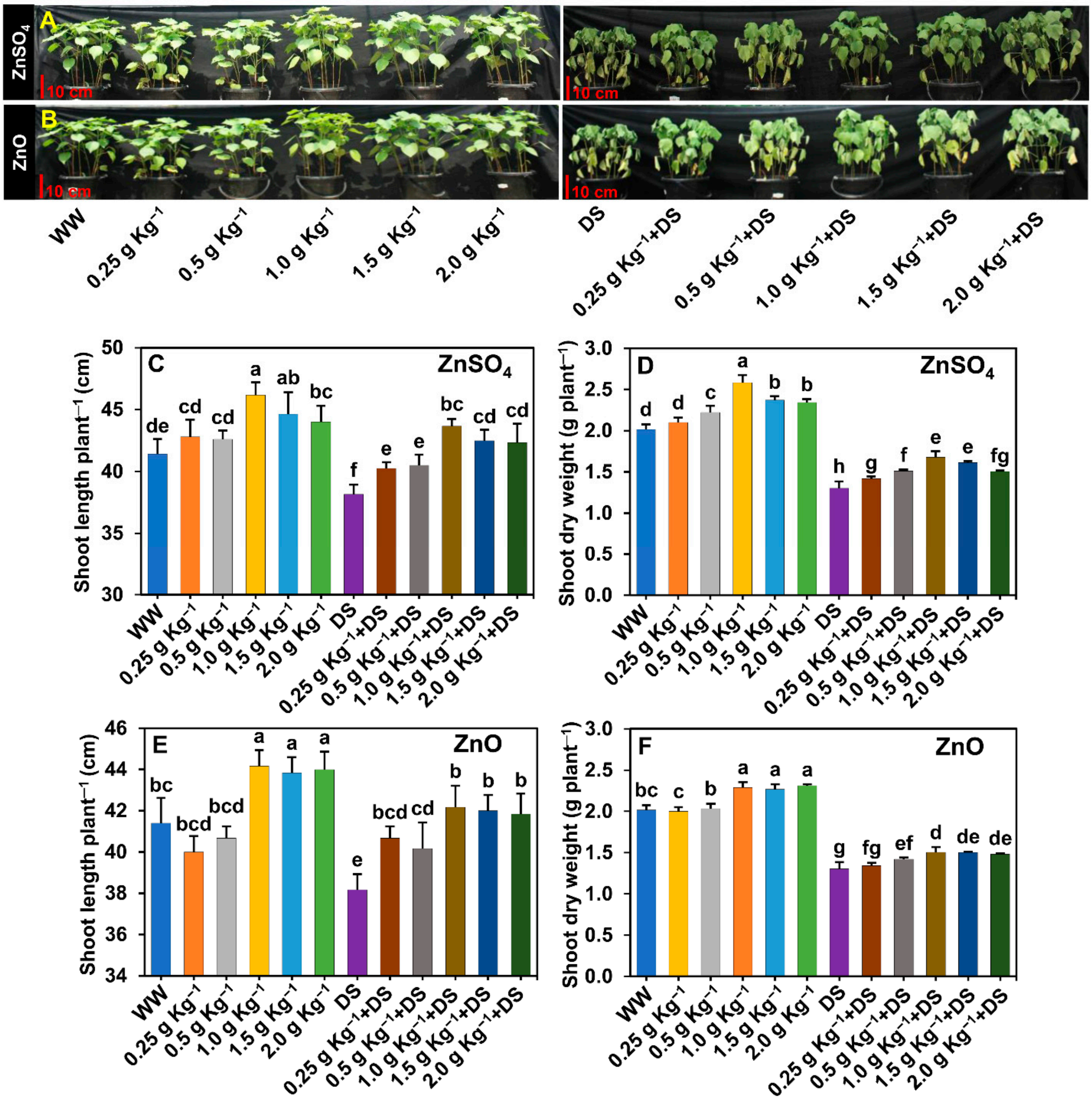

Figure S1. Effect of separately applied different doses of $ZnSO_4$ (0.25, 0.5, 1.0, 1.5, and 2.0 g Kg^{-1} soil) and ZnO (0.25, 0.5, 1.0, 1.5, and 2.0 g Kg^{-1} soil) on cotton plants that were subjected to water deprivation-induced drought stress for 6 days. (**A**,**B**) Cotton plants were photographed after being subjected to drought stress. (**C**) Shoot length and (**D**) shoot dry weight of cotton plants supplemented with different doses of $ZnSO_4$. (**E**) Shoot length and (**F**) shoot dry weight of cotton plants supplemented with different doses of ZnO. The means and standard deviations (n = 3) are displayed as bars. Significant changes ($p < 0.05$) among the treatments are denoted by different letters above the bars as calculated using the least significant difference test. WW, well-watered plants; DS, drought-stressed plants.
